# The evolution of host associations in the parasitic wasp genus *Ichneumon* (Hymenoptera: Ichneumonidae): convergent adaptations to host pupation sites

**DOI:** 10.1186/1471-2148-13-74

**Published:** 2013-03-27

**Authors:** Andreas Tschopp, Matthias Riedel, Christian Kropf, Wolfgang Nentwig, Seraina Klopfstein

**Affiliations:** 1 Natural History Museum, Department of Invertebrates, Bernastrasse 15, Bern, CH-3005, Switzerland; 2 Institut of Ecology and Evolution, Division of Community Ecology, University of Bern, Balzerstrasse 6, Bern, CH-3012, Switzerland; 3 Klinik Fallingbostel, Kolkweg 1, Bad Fallingbostel, D-29683, Germany; 4 Naturhistoriska Riksmuseet, Box 50007, Stockholm, SE-104 05, Sweden

**Keywords:** Idiobionts, Parasitoid wasp, Phylogeny, Homoplasy, Host relations

## Abstract

**Background:**

The diversification of organisms with a parasitic lifestyle is often tightly linked to the evolution of their host associations. If a tight host association exists, closely related species tend to attack closely related hosts; host associations are less stable if associations are determined by more plastic traits like parasitoid searching and oviposition behaviour. The pupal-parasitoids of the genus *Ichneumon* attack a variety of macrolepidopteran hosts. They are either monophagous or polyphagous, and therefore offer a promissing system to investigate the evolution of host associations. *Ichneumon* was previously divided into two groups based on general body shape; however, a stout shape has been suggested as an adaptation to buried host pupation sites, and might thus not represent a reliable phylogenetic character.

**Results:**

We here reconstruct the first molecular phylogeny of the genus *Ichneumon* using two mitochondrial (CO1 and NADH1) and one nuclear marker (28S). The resulting phylogeny only supports monophyly of *Ichneumon* when *Ichneumon lugens* Gravenhorst, 1829 (formerly in *Chasmias,* stat. rev.) and *Ichneumon deliratorius* Linnaeus, 1758 (formerly *Coelichneumon*) are included. Neither parasitoid species that attack hosts belonging to one family nor those attacking butterflies (Rhopalocera) form monophyletic clades. Ancestral state reconstructions suggest multiple transitions between searching for hosts above versus below ground and between a stout versus elongated body shape. A model assuming correlated evolution between the two characters was preferred over independent evolution of host-searching niche and body shape.

**Conclusions:**

Host relations, both in terms of phylogeny and ecology, evolved at a high pace in the genus *Ichneumon*. Numerous switches between hosts of different lepidopteran families have occurred, a pattern that seems to be the rule among idiobiont parasitoids. A stout body and antennal shape in the parasitoid female is confirmed as an ecological adaptation to host pupation sites below ground and has evolved convergently several times. Morphological characters that might be involved in adaptation to hosts should be avoided as diagnostic characters for phylogeny and classification, as they can be expected to show high levels of homoplasy.

## Background

The evolution of host ranges in parasitic life forms deserves special attention, not only because it allows the investigation of numerous questions central to evolutionary biology, but also because of the tremendous ecological and economic importance of ecosystem functions delivered by these species. The time-scales over which processes like host-switching and co-speciation take place are of immediate interest as they not only help us understand current host ranges, but also predict future developments and adaptability of parasitic species. Insect parasitoids represent a special case of parasitic organisms because they ultimately kill their hosts during development. They are often classified ecologically into idiobionts and koinobionts. Idiobionts prevent further development of the host after initially immobilizing it, while koinobionts allow the host to continue its development after parasitization, often over several host life stages [1,2]. While many koinobionts show high degrees of specialization and host fidelity, idiobionts are usually generalists and can even vary in their host ranges even at the population level. In such generalists, individuals often show a high level of behavioural and developmental plasticity as a response to an inconstant environment, and this plasticity can be crucial for their persistence [3]. On a macro-evolutionary level, such plasticity can result in a high rate of host switching. If host switches are common in the evolutionary history of a group, then the phylogenies of hosts and parasitoids show low concordance [4]. The opposite pattern, i.e., high concordance between host and parasitoid phylogenies, can result from very tight associations and a correspondingly low frequency of host switches, and in the extreme even co-speciation between host and parasites or parasitoids [5-7]. An intermediate level of phylogenetic concordance can be expected if host ranges evolve according to the “host-ecology hypothesis” [3,8-10]. This hypothesis assumes that parasitoid species are able to broaden their host ranges by recruiting new hosts that exist within the parasitoids searching niche, and that this process can eventually lead to the appearance of a new, specialist species. Specialization thus takes place on the level of the host’s niche instead of its taxonomic or phylogenetic identity.

In parasitoid wasps, our knowledge of host range evolution is very limited due to a lack both of reliable host records in many groups and of sound species-level phylogenies [1,11]. Very few studies have examined the evolution of host ranges and thus the prevalence of different macro-evolutionary processes from a phylogenetic perspective [8,12-15]. The specious parasitic wasp genus *Ichneumon* Linnaeus, 1758 (Hymenoptera: Ichneumonidae, Ichneumoninae) consists mainly of endoparasitoids that attack the pupal stage of their macro-lepidopteran hosts [16,17]. After parasitization, the hosts do not continue to grow and the parasitoid larvae thus have to develop on the host resources present at the time of oviposition; most *Ichneumon* species thus follow the idiobiont strategy of development [2]. Several exceptions however exist in the genus, e.g., *Ichneumon eumerus* Wesmael, 1857 and *Ichneumon caloscelis* Wesmael, 1845 that attack the larva of their hosts, while emerging from the pupa [18,19]. These species clearly are koinobionts and might show a closer association with their hosts. Within *Ichneumon*, some species are highly polyphagous as is typical for idiobionts, while other species are known only from a single host species [17]; this genus therefore offers an interesting system to study the evolution of host association patterns and host specificity.

**Figure 1:**
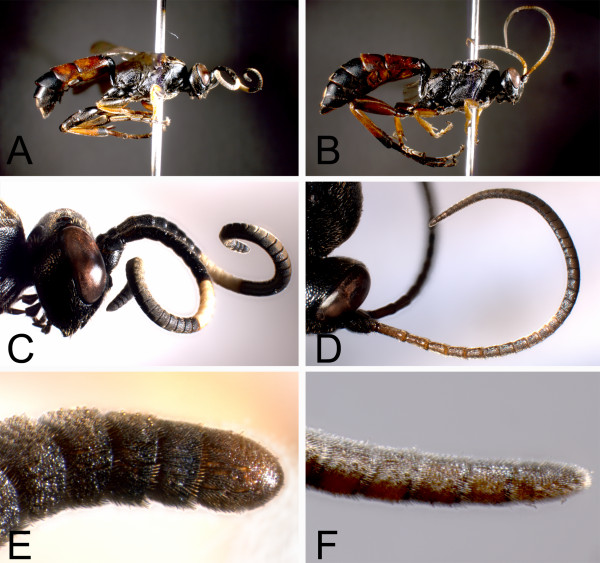
**Body shape and antennal shape of *Ichneumon extensorius *and *I. gracilicornis*.***I. extensorius***(A, C, E)** is a representative of species with a compact body shape, *I. gracilicornis***(B, D, F)** represents the group of slender body shapes. The antennae of the former are shortened, stout, and the apical antennomeres are broadened; the antennae of the latter are relatively long and with the terminal antennomeres longer than wide.

Based on morphological investigations and laboratory host choice experiments, Hinz and Horstmann [17] differentiated between two groups of species within the genus *Ichneumon*. The first group consists of polyphagous parasitoids that generally attack species of Noctuidae moths that pupate in cavities below ground. Hosts of the second group pupate above ground or in the vegetation; their parasitoids are more often oligophagous or even monophagous, and include many species that attack butterflies (Rhopalocera). Hilpert’s [16]*ad hoc* hypothesis of the phylogeny of the genus *Ichneumon* was based on the assumption that these two parasitoid groups represent natural entities. As possible synapomorphies for the two groups, he cited the overall body shape and especially the form of the antenna in the females, which are short and stout with a blunt tip in the first and elongated and pointed in the second group (Figure 1). The shortening of the antennae, which represent an overall more compact body shape, is discussed as an adaptation to searching for hosts that pupate below ground, where long antennae would be obstructive and prone to injuries [17]. Body and antennal shape might thus be misleading as phylogenetic characters in *Ichneumon*, as they might have appeared several times through convergent evolution by adaptation to host pupation sites [20-22]. Understanding the evolution of host relationships in a group can thus also be crucial for a proper interpretation of morphological characters in a phylogenetic context, as has been shown for parasitic wasps already several times in the past [23-25].

Here, we build the first molecular phylogeny of the genus *Ichneumon* including 38 species using two mitochondrial markers, cytochrome oxydase 1 (CO1) and NADH dehydrogenase 1 (NADH1), and the nuclear 28S rRNA (D2-D3 region). The molecular phylogeny was reconstructed using maximum likelihood (ML) and Bayesian approaches. To investigate whether parasitoids that attack host species of the same family cluster together, we plotted host family associations onto the parasitoids phylogeny. Additionally, we tested for monophyly of the butterfly parasitoids under a likelihood-based and a Bayesian approach. To test the host-ecology hypothesis for *Ichneumon*, the evolution of the parasitoids’ searching niche was reconstructed. Finally, we tested for correlated evolution between antennal shape and the host pupation site.

## Results

### Phylogenetic reconstruction

**Figure 2:**
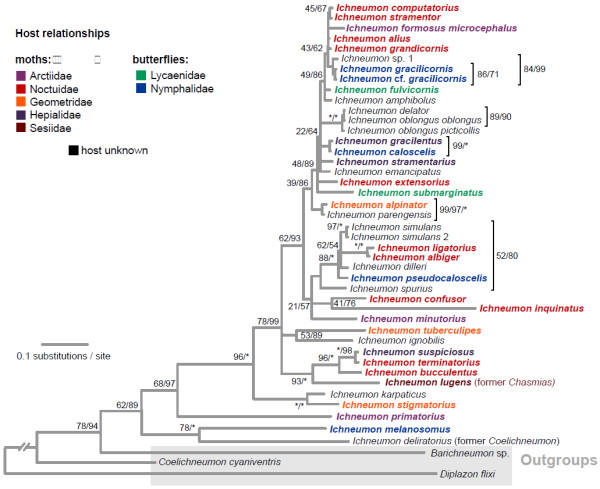
**Bayesian consensus tree of *Ichneumon *species showing host relationships.** Bayesian consensus tree based on 28S, CO1 and NADH1 sequences, partitioned by gene and codon position and using a 16-state Doublet model for the pairing positions of 28S. For each node, the support is given as maximum-likelihood bootstrap proportions and Bayesian posterior probabilities, with asterisks representing maximum support. Host families are indicated by colour codes for the *Ichneumon* species (see legend).

The Bayesian consensus tree recovered for the 38 *Ichneumon* and five outgroup taxa (Table 1) is shown in Figure 2. The topologies obtained from the maximum-likelihood and Bayesian analyses were highly congruent and conflicting nodes between the consensus trees only reached low support. Most of the relationships are resolved with high confidence, and species that were represented by more than one specimen were recovered as monophyletic. Some of the more recent nodes are however associated with very short branches and low support values. Our dataset did not provide any resolution for several more closely related species pairs, with identical CO1 barcodes encountered for *Ichneumon delator* and *I. oblongus* and for *I. gracilentus* and *I. caloscelis*, respectively. Pairwise uncorrected p-distances below 1% in CO1 were found for 21 additional pairs of species.

**Table 1:** Species, specimen numbers and origins, and Genbank accession numbers

**Taxon**	**Specimen**	**Country/Department/Locality/Collection date**	**CO1**	**NADH1**	**28S**
***Ichneumon***					
*Ichneumon albiger* Wesmael, 1845	at_17	SWITZERLAND/Nidwalden/Hergiswil, Alpgschwänd/22.03.2009	JX453396	JX453347	
*Ichneumon alius* Tischbein, 1879	at_12	SWITZERLAND/Graubünden/Sur, Alp Flix/16.06.2003	JX453384	JX453346	JX453422
*Ichneumon alpinator* Aubert, 1964	at_11	SWITZERLAND/Graubünden/Sur, Alp Flix/28.07.2003	JX453383	JX453341	JX453421
*Ichneumon amphiboles* Kriechbaumer, 1888	at_47	SWEDEN/Stochholms län/Haninge, Tyresta/21.07.2003	JX453412	JX453371	
*Ichneumon bucculentus* Wesmael, 1845	at_20	SWEDEN/Stockholms län/Södertälje, Tullgarn/19.08.2004	JX453392		
*Ichneumon caloscelis* Wesmael, 1845	at_42	SWEDEN/Kalmar län/Högsby, Hornsö kronopark/10.08.2003	JX453408	JX453367	JX453428
*Ichneumon computatorius* Müller, 1776	at_8	SWEDEN/Kalmar län/Nybro, Bäckebo/19.06.2005	JX453389	JX453339	JX453419
*Ichneumon confusor* Gravenhorst, 1820	at_7	SWEDEN/Kalmar län/Nybro, Alsterbo/10.06.2006	JX453388	JX453338	JX453418
*Ichneumon delator* Wesmael, 1845	at_4	SWEDEN/Västerbottens län/Vindeln, Kulbäckslidens försökspark/03.09.2004	JX453380	JX453337	JX453416
*Ichneumon dilleri* Heinrich, 1980	at_15	SWITZERLAND/Graubünden/Sur, Alp Flix/15.07.2006	JX453395	JX453350	
*Ichneumon emancipatus* Wesmael, 1845	at_46	SWEDEN/Uppsala län/Håbo, Biskops-Arnö/18.07.2005	JX453411	JX453370	
*Ichneumon extensorius* Linnaeus, 1758	at_18	SWITZERLAND/Luzern/Horw, Schwendelberg/12.03.2009	JX453390	JX453348	JX453431
*Ichneumon formosus microcephalus* Stephens, 1835	at_5	SWEDEN/Hallands län/Laholm, Mästocka/04.10.2003		JX453344	
*Ichneumon fulvicornis* Gravenhorst, 1829	at_31	SWEDEN/Västerbottens län/Vindeln, Kulbäckslidens försökspark/22.09.2003	JX453404	JX453363	
*Ichneumon gracilentus* Wesmael, 1845	at_30	SWEDEN/Kronobergs län/Älmhult, Stenbrohult/20.07.2005	JX453403	JX453362	
*Ichneumon gracilicornis* Gravenhorst, 1829	at_25	SWITZERLAND/Graubünden/Sur, Alp Flix/27.07.2006	JX453399	JX453354	
*Ichneumon cf. gracilicornis* Gravenhorst, 1829	at_33	SWITZERLAND/Bern/Lenk, Oberlaubhorn/10.07.2009	JX453406	JX453365	
*Ichneumon grandicornis* Thomson, 1886	at_28	SWEDEN/Hallands län/Halmstad, Gardshult/13.07.2005	JX453402	JX453361	JX453426
*Ichneumon ignobilis* Wesmael, 1855	at_48	SWEDEN/Västerbottens län/Vindeln, Kulbäckslidens försökspark/22.09.2003	JX453413	JX453372	JX453430
*Ichneumon inquinatus* Wesmael, 1845	at_9	SWITZERLAND/Nidwalden/Hergiswil, Alpgschwänd/22.03.2009	JX453387		
*Ichneumon karpaticus* Heinrich, 1951	at_24	SWEDEN/Norbottens län/Jokkmokk, Muddus nationalpark/18.06.2004	JX453398	JX453359	
*Ichneumon ligatorius* Thunberg, 1822	at_27	SWEDEN/Västerbottens län/Vindeln, Kulbäcken meadow/20.08.2004	JX453401	JX453360	
*Ichneumon melanosomus* Wesmael, 1855	at_21	SWEDEN/Gävleborgs län/Hudiksvall, Stensjön/11.08.2004	JX453393	JX453351	
*Ichneumon minutorius* Desvignes, 1856	at_23	SWEDEN/Stochholms län/Haninge, Tyresta/20.07.2004	JX453397	JX453353	
*Ichneumon oblongus oblongus* Schrank, 1802	at_32	SWEDEN/Kronobergs län/Älmhult, Stenbrohult/06.05.2004	JX453405	JX453364	
*Ichneumon oblongus picticollis* Holmgren, 1864	at_3	SWEDEN/Västerbottens län/Vindeln, Svartbergets försökspark/22.09.2003	JX453379	JX453336	
*Ichneumon parengensis* Kiss, 1929	at_39	SWITZERLAND/Graubünden/Sur, Alp Flix/21.06.2003	JX453407	JX453366	JX453427
*Ichneumon primatorius* Forster, 1771	at_14	SWITZERLAND/Graubünden/Sur, Alp Flix/15.07.2006	JX453386	JX453358	JX453424
*Ichneumon pseudocaloscelis* Heinrich, 1949	at_13	SWITZERLAND/Graubünden/Sur, Alp Flix/09.06.2003	JX453385	JX453357	JX453423
*Ichneumon simulans* Tischbein, 1873	at_1	SWEDEN/Kalmar län/Nybro, Bäckebo/18.05.2006	JX453377	JX453342	JX453414
*Ichneumon simulans* 2 Tischbein, 1873	at_10	SWITZERLAND/Nidwalden/Hergiswil, Alpgschwänd/22.03.2009	JX453382	JX453340	JX453420
*Ichneumon spurius* Wesmael, 1848	at_2	SWEDEN/Västa Götalands län/Stenungsund/25.05.2004	JX453378	JX453343	JX453415
*Ichneumon stigmatorius* Zetterstedt, 1838	at_45	SWEDEN/Västerbottens län/Vindeln, Kulbäckslidens försökspark/22.09.2003	JX453410	JX453369	JX453429
*Ichneumon stramentarius* Gravenhorst, 1820	at_19	SWITZERLAND/Luzern/Luzern, Allmend/04.03.2009	JX453391	JX453349	
*Ichneumon stramentor* Rasnitsyn, 1981	at_43	SWEDEN/Kronobergs län/Älmhult, Stenbrohult/01.11.2003	JX453409	JX453368	
*Ichneumon submarginatus* Gravenhorst, 1829	at_22	SWEDEN/Uppsala län/Älvkarleby, BatforSweden/01.07.2004	JX453394	JX453352	
*Ichneumon suspiciosus* Wesmael, 1845	at_26	SWEDEN/Skåne län/Klippans, Skäralid/06.08.2004	JX453400	JX453355	JX453425
*Ichneumon terminatorius* Gravenhorst, 1820	at_29	SWEDEN/Kronobergs län/Älmhult, Stenbrohult/01.08.2003		JX453356	
*Ichneumon tuberculipes* Wesmael, 1848	at_6	SWEDEN/Stockholms län/Haninge, Tyresta/20.07.2004	JX453381	JX453345	JX453417
*Ichneumon sp.* 1	Seb_6_8	France/Hautes-AlpeSweden/Col du Lautaret/summer 2008	GU597830	GU597771	GU597591
**Outgroups**					
*Barichneumon* sp.	at_34	SWITZERLAND/Bern/Bern, Bremgartenwald/18.08.2008	JX453373	JX453332	JX453373
*Coelichneumon cyaniventris* (Wesmael, 1859)	at_35	SWITZERLAND/Bern/Bern, Bremgartenwald/20.06.2008	JX453374	JX453333	JX453374
*Ichneumon deliratorius* Linnaeus, 1758 (former *Coelichneumon*)	at_41	SWEDEN/Stockholms län/Södertälje, Tullgarn/17.07.2005	JX453375	JX453334	JX453375
*Ichneumon lugens* Gravenhorst, 1829 (former *Chasmias*)	at_16	SWITZERLAND/Nidwalden/Hergiswil, Alpgschwänd/22.03.2009	JX453376	JX453335	
*Diplazon flixi* Klopfstein, 2013	SK_1A2	SWITZERLAND/Graubünden/Sur, Alp Flix/17.07.2006	FJ556425	GU597691	FJ556492

Maximum likelihood and Bayesian analyses all only support the monophyly of the genus *Ichneumon* when it is expanded to include *Chasmias lugens* and *Coelichneumon deliratorius*. The support for the monophyly of such an *Ichneumon* s. l. was high in both analyses (bootstrap support: 0.85, posterior probability: 0.89) (Figure 2), while monophyly of the genus excluding *C. lugens* and *C. deliratorius* proved to be very unlikely (SH test, p<0.001).

### Evolution of host ranges

Parasitoid species that attack hosts that belong to a single lepidopteran family do not cluster together, as shown in Figure 2, but instead appear in distant parts of the tree. Sister species often attack hosts from different families, and parasitoids of none of the host families were recovered as monophyletic. Also the parasitoids of butterfly hosts were recovered as paraphyletic in all our analyses, and the hypothesis of monophyly of these species was highly rejected both by a Bayesian approach (Bayes factor: 195.28) and by the Shimodaira-Hasegawa test [26] (p< 0.001).

**Figure 3:**
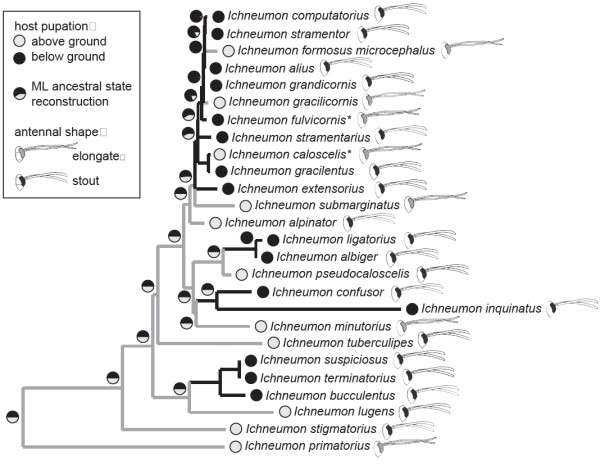
**Ancestral state reconstruction of host-searching niche.** Host searching niches are given for the terminal taxa together with antennal shape (see legend). In most species, the niches represent the pupation sites, while the whereabouts of the last instar caterpillars are given for the two larval-pupal parasitoids (marked with an asteriks). Maximum-likelihood ancestral-state reconstructions of the host-searching niches are given for each node, and maximum-parsimony reconstructions are shown as branch colours.

Species that attack their hosts above or below ground, respectively, do not form monophyletic clades either (Figure 3). Parsimony and maximum likelihood ancestral state reconstructions revealed that this behavioural trait changed at least five times during the evolution of the genus *Ichneumon*. This is the case when all the nodes that received low support are resolved in order to minimize the number of switches; in the consensus topology, this character showed at least seven state changes. Reconstructing the character states at the deeper nodes of the phylogeny proved virtually impossible for such a fast-evolving character, and although attacking hosts that pupate above ground was favored as the ancestral state for the genus, this result was not obtained under ML, and might be highly dependent on the taxon sampling.

### Morphological adaptations to host pupation site

To test for correlated evolution between parasitoid body shape (Figure 1) and attacking hosts with specific pupation sites (Table 2), we compared the likelihoods of a model assuming independent evolution to a model assuming that the two traits coevolved [27]. The likelihood ratio test significantly supports the dependent model (LHR= 10.55, df= 4, p= 0.032). This result was confirmed by the Bayesian approach which converged on the dependent model with 99.63% posterior probability (Bayes factor: 3.26). Although the correlation was generally supported, there are several exceptions to this rule (*I. alpinator*, *I. fulvicornis* Gravenhorst, 1829, *I. pseudocaloscelis* Heinrich, 1949, *I. stigmatorius* Zetterstedt, 1838, *I. tuberculipes* Wesmael, 1848, and *C. lugens*, see Figure 3). Except for *I. fulvicornis* which has an elongated antennae but attacks hosts pupating below ground, these species have a more compact body shape, but attack hosts above ground.

**Table 2:** ***Ichneumon *species, their antennal shape and host pupation site**

**Species**	**Antenna**	**Host pupation**
*Ichneumon albiger*	short	below ground
*Ichneumon alius*	short	below ground
Ichneumon alpinator*	short	above ground
*Ichneumon amphibolus*	short	-
Ichneumon bucculentus*	short	below ground
*Ichneumon caloscelis#*	long	above ground
*Ichneumon cf. gracilicornis*	long	above ground
*Ichneumon computatorius*	short	below ground
*Ichneumon confusor*	short	below ground
*Ichneumon delator*	-	-
*Ichneumon dilleri*	short	-
*ichneumon emancipatus*	long	-
*Ichneumon extensorius*	short	below ground
Ichneumon formosus microcephalus*	long	above ground
Ichneumon fulvicornis*#	long	below ground
Ichneumon gracilentus*	short	below ground
*Ichneumon gracilicornis*	long	above ground
*Ichneumon grandicornis*	short	below ground
*Ichneumon ignobilis*	short	-
*Ichneumon inquinatus*	short	below ground
*Ichneumon karpaticus*	short	-
*Ichneumon ligatorius*	short	below ground
*Ichneumon lugens*	short	above ground
*Ichneumon minutorius*	long	above ground
*Ichneumon oblongus*	short	-
*Ichneumon oblongus picticollis*	short	-
*Ichneumon parengensis*	short	-
Ichneumon primatorius*	long	above ground
Ichneumon pseudocaloscelis*	short	above ground
*Ichneumon simulans*	short	-
*Ichneumon simulans* 2	short	-
*Ichneumon spurius*	short	-
Ichneumon stigmatorius*	short	above ground
*Ichneumon stramentarius*	short	below ground
*Ichneumon stramentor*	short	below ground
*Ichneumon submarginatus*	long	above ground
Ichneumon suspiciosus*	short	below ground
*Ichneumon terminatorius*	short	below ground
Ichneumon tuberculipes*	short	above ground
*Ichneumon sp. 1*	long	-

*Species marked with an asterisk are known only from a single host species, and are thus potentially monophagous.

#*Ichneumon caloscelis* and *Ichneumon fulvicornis* attack the larvae of their hosts, and the habitat of the last instar larvae are here given instead of the pupation site.

## Discussion

### Phylogeny of *Ichneumon* and implications for taxonomy

We here present the first molecular phylogeny of the genus *Ichneumon*. It will serve as a robust starting point for future investigations of this specious genus, both in terms of phylogenetic and evolutionary research. Our molecular dataset provided good resolution of most of the nodes in the tree, but proved not to be variable enough to resolve some of the more recent relationship. Even the mitochondrial locus used for DNA barcoding [28], cytochrome oxidase 1, did not allow distinguishing among all the included *Ichneumon* species, with identical barcodes observed at least in two species pairs, and pairwise distances below 1% in many more. A similar observation has been made in the ichneumonid subfamily Diplazontinae [29,30], but CO1 has proven very useful in other groups of parasitic wasps [13,31]. The failure of DNA barcoding in *Ichneumon* might be due to imperfect taxonomy, insufficient variability of the markers to detect relatively recent speciation events, or in some of the cases due to incomplete lineage sorting or introgression [32,33]. More data, including several fast-evolving nuclear markers like introns will probably be necessary, as non-monophyly of biological species in mitochondrial DNA has been convincingly demonstrated already in several cases, and might concern up to a third of all species in nature [34,35].

The genus *Ichneumon* as it is currently defined was not retrieved as monophyletic (Figure 2), unless *Chasmias lugens* and *Coelichneumon deliratorius* were included. The relations of these species to *Ichneumon* have been discussed controversially in the past, and the morphological definition of the genus is based mainly on characters that might well be plesiomorphic [16,17,36]. *Chasmias lugens* does not fit well into the genus *Chasmias* morphologically, and based on our result should definitely be treated as part of the genus *Ichneumon*, where we transfer it hereby (stat. rev.). *Coelichneumon deliratorius*, based on morphology and on the results of the current study, has recently been re-included in the genus *Ichneumon*[37].

The molecular phylogeny recovered here clearly refutes the *ad hoc* hypothesis of the evolution of this genus as it was put forward by Hilpert [16]. The synapomorphies that Hilpert suggested to support his cladogram are mostly mere trends [15] and included several character states that are putative adaptations to parasitizing particular hosts. As one example, Hilpert used a stout versus elongated shape of the female body and antennae to support an early split within *Ichneumon*. We could demonstrate here that a stout body shape is probably an adaptation to searching for hosts below ground. Character states associated with host relations can be misleading for classification and phylogenetic reconstruction, as has been shown for various groups of parasitoid wasps [21-25,38-40]. In brief, such characters are only reliable if the switch to a particular host group happened only once during the evolutionary history of a group of parasitoids, but are prone to be homoplasious if it has been colonized several times in parallel.

### Numerous switches between host families and between host searching niches

Host ranges in *Ichneumon* have undergone numerous switches during the evolution of this genus, and there was no sign of a conservative evolution of host associations among the species examined here (Figure 2). On the other hand, the *Ichneumon* species known to be polyphagous are usually restricted to hosts from a single family, demonstrating specialization at a low taxonomic level. Our taxon sampling was too sparse to predict how often host families are retained across speciation events, even though some of the included species might be closely related. Reliable host records are only available for a small fraction of the known *Ichneumon* species, and well-identified material suited for DNA extraction is difficult to get. The 38 species sampled here only represent a small fraction of the total species diversity of the genus, and if minor radiations have taken place within a host group, they might have been overlooked with our limited taxon sampling. In any case, our study provides a conservative estimate of the minimum number of host switches that took place during the evolution of this genus.

Although similar studies are scarce, a prominent role for host switching in shaping the host ranges of parasitoid wasps has been demonstrated in several cases. Sime & Wahl (ref. 2002), based on a morphological phylogeny, observed separate origins of butterfly parasitism in the *Callajoppa* genus group (Ichneumonidae, Ichneumoninae), and stated that host ranges in these parasitoids were dominated by comparatively recent host switches. A similar scenario was put forward by Shaw [14], again based on a morphological phylogeny. Zaldivar-Riveron et al. [8] used molecular markers in combination with a calibrated relaxed clock analysis to show that host associations changed quickly during the evolution of rogadinae braconids, and that the radiation of the wasps took place dozens of millions of years after the radiation of their hosts.

In terms of the niche where *Ichneumon* females search for their hosts, we observe a similar pattern. Polyphagous species only attack hosts that can be found either above or below ground, but no conservatism was apparent on a higher phylogenetic level (Figure 3), as it would be predicted under the host-ecology hypothesis [3,8,41]. Again, our taxon sampling does not exclude the possibility of smaller radiations within one searching niche, as it has been demonstrated for the braconid wasp genus *Aleiodes*[8]. In this genus, closely related species tend to parasitize hosts with similar physical and ecological properties but which do not need to be closely related.

A high level of behavioural plasticity in host searching and host selection could be an explanatory factor for the macro-evolutionary patterns that we observed here, especially as behavioural traits have been shown to be less stable than physiological or morphological traits on evolutionary time-scales [42,43]. Shaw [3] suggested that a new host association resulting from behavioural plasticity of a female parasitoid wasp might even be passed on to its progeny through post-eclosion or pre-adult experience [44,45]. These mechanisms could enable the parasitoids to respond quickly to changes in host availability. They might be especially important in idiobiont parasitoids that only spend a short period of time in close association with the living host and thus do not need to adapt as much to the host’s physiological environment as koinobionts. Anecdotal evidence for the importance of host searching behaviour in comparison to host physiology stems from a laboratory experiment with *Ichneumon hinzi*, a supposedly monophagous parasitoid of *Xestia speciosa* (Hübner, 1813). In the laboratory, the parasitoid females also accepted the pupae of other, not closely related noctuids, and their progeny could successfully complete development in these non-host species [17]. These hosts are probably excluded from the natural host range of *I. hinzi* through a narrow search strategy of the female that is focussed on its primary host.

## Conclusions

We here present evidence that the evolution of host ranges in the parasitoid wasp genus *Ichneumon* included multiple transitions between host families and between microhabitats where the hosts can be found. Similar studies are scarce due to a lack of well-supported phylogenies for most groups and, more importantly, a lack of reliable host records for most parasitoid species. New molecular techniques, e.g., the DNA barcoding of host and parasitoid remains, or even of the gut contents of adult parasitoid wasps [46], might in the future complement time-intensive field observations and rearing as a means to document host-parasitoid associations and will thus allow for a more detailed picture of the evolution of host ranges in *Ichneumon* as well as in other parasitoid wasps. A better understanding of the dynamics and speed of the evolution of host associations will be crucial in order to predict adaptability of parasitoids to changes in the environment. Furthermore, it has important implications for risk assessments in bio-control, and for the comprehension of the tremendous diversity of parasitoid wasps.

## Methods

### Taxon sampling

We included 40 individuals of 38 *Ichneumon* species and subspecies in our study (Table 1). For two species, we sequenced two individuals for different reasons. A male of *I. gracilicornis*, a species that can only be determined with certainty in the female sex, was added to check the identification. Second, two *I. simulans* females showed large size differences and were collected in different countries. The genus *Ichneumon* is defined by a number of plesiomorphic characters, but also by several probably derived characters [16]. *Chasmias lugens* was in the past variously combined with the genera *Ichneumon* or *Chasmias*. Because morphologically, it takes a rather isolated position within *Chasmias*, we also included it in our analysis. Moreover, *Coelichneumon deliratorius* shares several morphological and colour traits with *Ichneumon* species, but does not hibernate as an adult [47], which represents the only marked difference from *Ichneumon*. This species was also included in our analysis to investigate its phylogenetic position. As outgroups, we included representatives from the genera *Barichneumon* and *Coelichneumon* from the same subfamily, and the more distantly related *Diplazon* as a functional outgroup. We could obtain sequences of 42, 43 and 20 individuals from the markers NADH1, CO1 and 28S, respectively, and Genbank accession numbers are given in Table 1.

### Molecular methods

The specimens used were either preserved in 80% ethanol or air dried. Genomic DNA was extracted either from whole insects or, if the specimens were larger than 1.5 cm, from the metasoma, using the Promega Wizard kit for blood and tissue extraction. DNA samples are kept at the Natural history Museum in Bern (NMBE), vouchers at NMBE and at the Naturhistoriska Riksmuseet in Stockholm (NRM) (Table 1). Approximately 600 base pairs (bp) from the 5′ end of the mitochondrial CO1 gene were amplified using the primers designed by Folmer et al. [48]. From NADH1, the second mitochondrial gene, we amplified 390 bp using the primers described by Smith et al. [49]. To obtain about 650 bp of the nuclear 28S rRNA, the D2 and partial D3 region were amplified utilising primers designed by Belshaw and Quicke [50] and Mardulyn and Whitfield [51].

Polymerase chain reactions (PCR) were done in 20 μl final volumes using Promega GoTaq Flexi DNA Polymerase kits. Final volumes contained 30 pmol MgCl_2_, 16 pmol of each primer, 4 pmol of each dNTP, 0.3 U Taq polymerase and 2 μl genomic DNA. PCR conditions were: 94°C for 5 min, 37 cycles of 30s at 94°C, 30s at the respective annealing temperature (51°C for CO1, 48°C for NADH1 and 52°C for 28S), and 45 s at 72°C. PCR products were purified by the purification service of Macrogen Korea. The PCR products were sequenced on an ABI 377 automated sequencer using Big Dye Terminator technology (Applied Biosystems). Half of the taxa showed superimposed parts of the 28S sequences, probably due to the existence of different alleles due to incomplete concerted evolution of the ribosomal DNA; they were excluded from the analyses. The remaining 28S sequences are distributed over the whole tree and provided good resolution of the backbone, which is why we decided to include them despite a high level of missing data.

The sequences of the two protein-coding genes (CO1 and NADH1) were aligned after translation into amino acids using CLUSTAL [52] as implemented in Mega 4.0 [53] with default settings. For both genes, no indels were detected. The D2-D3 region of the large subunit of 28S rRNA was aligned according to published secondary structure maps of ichneumonids [54], identifying the stem regions for partitioning and the pairing nucleotide position for the application of the doublet model in MrBayes and RAxML (see below). Of the identified non-pairing regions, only those that were length-conserved across the alignment were included in the analyses, while length-variable stretches were excluded. We thus obtained a 616 bp fragment of CO1, a 389 bp fragment of NADH1and 571 unambiguously alignable basepairs of 28S. Variability patterns of the different molecular partitions were obtained from PAUP* [55], where we also conducted a test for compositional heterogeneity. As none of the partitions showed significant heterogeneity, we proceeded to analyse the data under homogeneous models of nucleotide substitution (see next paragraph).

### Phylogenetic analyses

Phylogenetic reconstructions were conducted using maximum-likelihood (ML) and Bayesian methods on the combined molecular data. We identified the best-fitting nucleotide substitution models for each partition using MrModeltest version 2.2 [56], with a neighbour-joining tree as the test tree and applying the Akaike information criterion [57]. The results of the model choice are shown in Table 3. Except for the 28S stem and 28S loop partitions, all partitions showed the best fit with models that incorporate rate heterogeneity across sites (Г or I+ Г). We tested different partitioning strategies according to the method proposed by Brandley et al. [58] and advocated by Brown and Lemmon [59]. Partitioning schemes are summarized in Table 4 and ranged from an unpartitioned analysis (P1) to a distinction of six partitions chosen based on gene identity and prior knowledge of biochemical properties (P6*): the pairing stem regions of 28 S, its remaining loop regions, combined first and second codon positions of each of the mitochondrial genes and finally third codon position of the mitochondrial genes. To obtain an estimate for the Bayes factors associated with each comparison of partitioning strategies, we conducted a Bayesian MCMC analysis on MrBayes v. 3.1.2 [60] for each strategy separately. Analyses were run with two independent runs of four chains each (heating T= 0.1), random starting trees and trees sampled every 1000 generations for at least 1*10^7^ generations. Convergence of the two runs was checked in mutliple ways. The log-likelihood scores (lnL) were plotted over generations and stabilisation determined. The overlay plot of the two independent runs was examined for a good mixing of the runs and stabilisation of the lnL. Then, we checked whether the standard deviation of split frequencies between the two runs fell below the 0.01 threshold [60]. Finally, we studied the behaviour of the potential scale reduction factor (PSRF) for the model parameters and clade supports, and considered the runs to have converged if the PSFR was less than 5% divergent from 1. We then conservatively discarded half of the generations as a burn-in, and obtained estimates of the marginal likelihood for Bayes factor comparisons from the harmonic means of the likelihood scores from the remaining generations using the sump command implemented in MrBayes. Convergence diagnostics revealed low convergence even after 1*10^7^ generations in the case of partitioning strategies P5 and P6. Although the lnL plot seemed to reach a plateau already after 10^7^ generations, and the overlay plot of the two runs revealed that they both stabilized on the same peak, the average split frequency did not decrease below 0.01 until generation 1.15*10^7^ in the case of P5 and oscillates around 0.01 in the case of P6. A new analysis with heating set to T=0.05 and the number of generations to 5*10^7^ did not produce convergence either. We think that the reason for this unusual convergence behaviour lies in the misspecification of the model that can cause the MCMC search to fail to converge for a long time period [30,61]. The likelihood scores of all the runs of P5 and P6 were distinctly below the value reached by the preferred P6* model (Table 4), and we thus did not further consider these partitioning strategies. For both the mitochondrial dataset alone and the three-genes approach, full partitioning was preferred by Bayes factor comparison. The less partitioned models can be rejected with high confidence in all cases, a pattern already observed in other partitioned Bayesian analysis [58,62].

**Table 3:** Properties of molecular partitions

**Partition**	**#bp**	**#var**	**#pars**	**#taxa**	**Model**
CO1	616	228	155	44	GTR+I+Г
CO1 first and second codon positions	410	74	39	44	GTR+I+Г
CO1 third codon positions	206	154	116	44	GTR+Г
NADH1	389	187	110	43	GTR+I+Г
NADH1 first and second codon positions	259	88	37	43	GTR+Г
NADH1 third codon postitions	130	99	73	43	HKY+Г
28S	571	57	6	19	GTR+Г
28S stem	354	31	4	19	GTR
28S loop	217	26	2	19	SYM
all markers combined	1576	493	279	46	GTR+I+Г

Abbreviations:

#bp. Number of base pairs.

#var. Number of variable sites.

#pars. Number of parsimony-informative sites.

#taxa. Number of terminals sequenced for the respective gene.

**Table 4:** Comparison of partitioning strategies

**Strategy**	**#part**	**Specification**	**lnL**	**lnBF**
P1	1	unpartitioned dataset	-8258.76	831
P2	2	partitioned according to mitochondrial (CO1 and NADH1) and nulclear (28S) gene identity	-7979.18	552
P3a	3	28S unpartitioned, mitochondrial markers partitioned into first and second versus third codon position	-7732.43	305
P3b	3	partitioned according to gene identity (CO1, NADH1 and 28S)	-7963.23	536
P5	5	28S unpartitioned, mitochondrial markers separately partitioned into first and second versus third codon position	-7563.92	137
P6	6	mitochondrial genes partitioned as under P5 and 28S partitioned into stem and loop	-7574.96	148
P6*	6	as P6, but with doublet model for the pairing stem partition of 28S	-7427.27	-

Abbreviations:

#part. Number of partitions.

lnBF. ln (Bayes factor).

The final likelihood analysis of the joint dataset was conducted using RaxML [63] under a GTR+Г+I model with 1,000 nonparametric bootstrap iterations, adopting the partitioning strategy preferred by Bayes factor comparisons and using a 16-state secondary structure model for the stem regions of 28S. Final Bayesian analyses were run for 2*10^7^ generations, and convergence was assessed as above. The matrix and resulting trees are deposited on TreeBASE http://purl.org/phylo/treebase/phylows/study/TB2:S13911.

### Evolution of host ranges

We obtained information on host families for the included *Ichneumon* species from the literature [17,64] and mapped them onto the consensus tree resulting from the Bayesian analysis (Figure 2) to look for host switching events. The five known butterfly parasitoids included in this study were recovered as paraphyletic in all our analyses. To test if this non-monophyly is statistically supported, we used a Bayes-factor and a likelihood-based approach. For the first, we conducted another Bayesian MCMC analysis, but imposing monophyly of the butterfly parasitoids as a phylogeny constraint, and compared the resulting marginal likelihood as estimated by the harmonic means. In addition, we applied the Shimodaira-Hasegawa test [26] as implemented in PAUP* [55] to the two maximum-likelihood phylogenetic hypotheses obtained with and without imposing the monophyly constraint.

### Morphological adaptation to hosts that are attacked below ground

To investigate the evolution of searching niches, we scored all species for the pupation sites of their hosts [65-67]. We distinguished between hosts pupating below ground, i.e. among plant roots or in the soil, and species whose pupae can be found above the ground, e.g., in the vegetation or fully exposed. For the larval-pupal parasitoid *I. caloscelis* that attacks the caterpillars of its host well before feeding has finished [19], the search habitat is certainly above ground where the hosts can be found feeding and resting, although one of its five known hosts, *Hipparchia semele*, pupates below ground. *I. fulvicornis* has been reared from *Phenagria* caterpillars found in ant nests. It is not entirely clear whether already the young caterpillars are attacked prior to the adoption by ants, i.e. above ground, but seems more likely that the female searches for last-instar caterpillars in the ant nests like *I. eumerus*[17]. We thus scored these two species according to the place where the last-instar larvae are found. We used parsimony and maximum likelihood to reconstruct ancestral states in the Ape package of the R statistical environment [68,69]. To test for correlated evolution of parasitoid body shape and hosts pupation sites, we used BayesDiscrete from the BayesTraits package [70], comparing a model of independent with one assuming dependent evolution. Likelihoods obtained under the two models with 50 ML attempts per tree were compared by a likelihood ratio test. Posterior probabilities of the dependent and independent models and harmonic means of the likelihoods for Bayes-factor comparison were obtained by Markov-chain Monte Carlo approaches. For this calculation, we applied an exponential reversible-jump hyperprior within the interval between zero and 30 and set the ratedev parameter that controls the proposal rate of new values, to 8. This resulted in an acceptance rate between 20% and 40%, which falls inside the recommended range [70].

## Competing interests

The authors declare that they have no competing interests.

## Authors’ contributions

SK, AT and WN planned this study. AT and SK conducted the data collection, identified part of the *Ichneumon* species, generated DNA sequences, and conducted the analyses of phylogeny and character-evolution. MR identified the largest part of the *Ichneumon* species and assisted with the interpretation of host ranges. CK, SK and AT contributed to the discussion of results and to the interpretation of the phylogeny and character evolution. All authors revised the manuscript drafts, read and approved the final manuscript.

## References

[B1] Quicke DLJ Parasitic Wasps 1997 London: Chapman and Hall

[B2] Askew RR Shaw MR Waage JK, Greathead D Parasitoid communities: Their size, structure and development Insect Parasitoids 1986 London: Academic 225 264

[B3] Shaw MR Hawkins BA, Sheehan W Parasitoid host ranges Parasitoid Community Ecology 1994 Oxford: Oxford University Press 111 144

[B4] Althoff DM A test of host-associated differentiation across the ‘parasite continuum’ in the tri-trophic interaction among yuccas, bogus yucca moths, and parasitoids Mol Ecol 2008 17 17 3917 3927 10.1111/j.1365-294X.2008.03874.x 18662219

[B5] Page RDM Tangled Trees. Phylogeny, Cospeciation and Coevolution 2002 Chicago: University of Chicago Press

[B6] Kikuchi Y Hosokawa T Nikoh N Meng X-Y Kamagata Y Fukatsu T Host-symbiont co-speciation and reductive genome evolution in gut symbiotic bacteria of acanthosomatid stinkbugs BMC Biol 2009 7 2 10.1186/1741-7007-7-2 19146674 PMC2637841

[B7] Hughes J Kennedy M Johnson KP Palma RL Page RDM Multiple cophylogenetic analyses reveal frequent cospeciation between Pelecaniform birds and *Pectinopygus* lice Syst Biol 2007 56 2 232 251 10.1080/10635150701311370 17464880

[B8] Zaldivar-Riverón A Shaw MR Sáez AG Mori M Belokobylskij SA Shaw SR Quicke DLJ Evolution of the parasitic wasp subfamily Rogadinae (Braconidae): phylogeny and evolution of lepidopteran host ranges and mummy characteristics BMC Evol Biol 2008 8 329 10.1186/1471-2148-8-329 19055825 PMC2614994

[B9] Shaw MR Melika G, Thuroczy C Host ranges of *Aleiodes* species (Hymenoptera: Braconidae), and an evolutionary hypothesis Parasitic Wasps: Evolution, Systematics, Biodiversity and Biological Control 2002 Budapest: Agroinform 321 327

[B10] Shaw MR Horstmann K An analysis of host range in the *Diadegma nanus* group of parasitoids in Western Europe, with a key to species (Hymenotpera: Ichneumonidae: Campopleginae) J Hym Res 1997 6 2 273 296

[B11] Quicke DLJ We know too little about parasitoid wasp distributions to draw any conclusions about latitudinal trends in species richness, body size and biology PLoS One 2012 7 2 e32101 10.1371/journal.pone.0032101 22355411 PMC3280234

[B12] Kankare M Stefanescu C van Nouhuys S Shaw MR Host specialization by *Cotesia* wasps (Hymenoptera: Braconidae) parasitizing species-rich Melitaeini (Lepidoptera: Nymphalidae) communities in north-eastern Spain Biol J Linn Soc Lond 2005 86 45 65 10.1111/j.1095-8312.2005.00523.x

[B13] Smith MA Rodriguez JJ Whitfield JB Deans AR Janzen DH Hallwachs W Hebert PDN Extreme diversity of tropical parasitoid wasps exposed by iterative integration of natural history, DNA barcoding, morphology, and collections Proc Natl Acad Sci USA 2008 105 35 12359 12364 18716001 10.1073/pnas.0805319105 PMC2518452

[B14] Shaw SR Euphorine phylogeny: the evolution of diversity in host-utilization by parasitoid wasps (Hymenoptera: Braconidae) Ecol Entomol 1988 13 323 335 10.1111/j.1365-2311.1988.tb00363.x

[B15] Sime KR Wahl DB The cladistics and biology of the *Callajoppa* genus-group (Hymenoptera: Ichneumonidae, Ichneumoninae) Zool J Linn Soc 2002 134 1 1 56 10.1046/j.1096-3642.2002.00006.x

[B16] Hilpert H Zur Systematik der Gattung Ichneumon Linnaeus, 1758 in der Westpalaearktis (Hyemnoptera, Ichneumonidae, Ichneumoninae) Entomofauna 1992 13 Suppl. 6 1 389

[B17] Hinz R Horstmann K Über Wirtsbeziehungen europäischer *Ichneumon*-Arten (On the host relationships of European species of *Ichneumon* Linnaeus (Insecta, Hymenoptera, Ichneumonidae, Ichneumoninae) Spinxiana 2007 30 1 39 63

[B18] Hochberg ME Elmes GW Thomas JA Clarke RT Effects of habitat reduction on the persistence of *Ichneumon eumerus* (Hymenoptera: Ichneumonidae), the specialist parasitoid of *Maculinea rebeli* (Lepidoptera: Lycaenidae) J Insect Conserv 1998 2 59 66 10.1023/A:1009644807126

[B19] Shaw MR On the distribution of some Satyrid (Lep.) larvae at a coastal site in relation to their Ichneumonid (Hym.) parasite Entomol Gaz 1977 28 2 133 134

[B20] van Noort S Compton SG Convergent evolution of agaonine and sycoecine (Agaonidae, Chalcidoidea) head shape in response to the constraints of host fig morphology J Biogeogr 1996 23 4 415 424 10.1111/j.1365-2699.1996.tb00003.x

[B21] Vilhelmsen L Head capsule concavities accommodating the antennal bases in hymenoptera pupating in wood: Possible emergence-facilitating adaptations Int J Insect Morphol 1997 26 2 129 138 10.1016/S0020-7322(97)00003-2

[B22] Vilhelmsen L Turrisi GF Per arborem ad astra: Morphological adaptations to exploiting the woody habitat in the early evolution of Hymenoptera Arthropod Struct Dev 2011 40 2 20 10.1016/j.asd.2010.10.001 20951828

[B23] Gauld ID Mound LA Homoplasy and the delineation of holophyletic genera in some insect groups Syst Entomol 1982 7 1 73 86 10.1111/j.1365-3113.1982.tb00127.x

[B24] Laurenne NM Karatolos N Quicke DLJ Hammering homoplasy: multiple gains and losses of vibrational sounding in cryptine wasps (Insecta: Hymenoptera: Ichneumonidae) Biol J Linn Soc Lond 2009 96 82 102

[B25] Quicke DLJ Belshaw R Incongruence between morphological data sets: an example from the evolution of endoparasitism among parasitic wasps (Hymenoptera: Braconidae) Syst Biol 1999 48 3 436 454 10.1080/106351599260094

[B26] Shimodaira H Hasegawa M Multiple comparisons of log-likelihoods with applications to phylogenetic inference Mol Biol Evol 1999 16 8 1114 1116 10.1093/oxfordjournals.molbev.a026201

[B27] Pagel M Meade A Bayesian analysis of correlated evolution of discrete characters by reversible-jump Markov chain Monte Carlo Am Nat 2006 167 6 808 825 10.1086/503444 16685633

[B28] Hebert PDN Ratnasingham S deWaard JR Barcoding animal life: cytochrome c oxidase subunit 1 divergences among closely related species Proc R Soc Lond B Biol Sci 2003 270 S96 S99 10.1098/rsbl.2003.0025 PMC1698023 12952648

[B29] Klopfstein S Quicke DLJ Kropf C Frick H Molecular and morphological phylogeny of Diplazontinae (Hymenoptera, Ichneumonidae) Zool Scr 2011 40 379 402 10.1111/j.1463-6409.2011.00481.x

[B30] Klopfstein S Kropf C Quicke DLJ An evaluation of phylogenetic informativeness profiles and the molecular phylogeny of Diplazontinae (Hymenoptera, Ichneumonidae) Syst Biol 2010 59 2 226 241 10.1093/sysbio/syp105 20525632

[B31] Stigenberg J Ronquist F Revision of the Western Palearctic Meteorini (Hymenoptera, Braconidae), with a molecular characterization of hidden Fennoscandian species diversity Zootaxa 2011 3084 1 95

[B32] Ballard JWO Rand DM The population biology of mitochondrial DNA and its phylogenetic implications Annu Rev Ecol Evol Syst 2005 36 621 642 10.1146/annurev.ecolsys.36.091704.175513

[B33] Galtier N Nabholz B Glémin S Hurst GDD Mitochondrial DNA as a marker of molecular diversity: a reappraisal Mol Ecol 2009 18 22 4541 4550 10.1111/j.1365-294X.2009.04380.x 19821901

[B34] Funk DJ Omland KE Species-level paraphyly and polyphyly: frequency, causes, and consequences, with insights from animal mitochondrial DNA Annu Rev Ecol Evol Syst 2003 34 397 423 10.1146/annurev.ecolsys.34.011802.132421

[B35] Bergsten J Bilton DT Fujisawa T Elliott M Monaghan MT Balke M Hendrich L Geijer J Herrmann J Foster GN The effect of geographical scale of sampling on DNA barcoding Syst Biol 2012 61 5 851 869 10.1093/sysbio/sys037 22398121 PMC3417044

[B36] Perkins JF Hymenoptera. Ichneumonoidea. Ichneumonidae, subfamilies Ichneumoninae II, Alomyinae, Agriotypinae and Lycorininae Handbooks for the identification of British insects. vol. VII. Part 2 (aii) 1960 London: Royal Entomological Society of London 117 213

[B37] Riedel M Revision der westpaläarktischen Arten der Gattung *Coelichneumon* THOMSON (Hymenoptera: Ichneumonidae: Ichneumoninae) Linz Biol Beitr 2012 44 2 1477 1611

[B38] Belshaw R Grafen A Quicke DLJ Inferring life history from ovipositor morphology in parasitoid wasps using phylogenetic regression and discriminant analysis Zool J Linn Soc 2003 139 2 213 228 10.1046/j.1096-3642.2003.00078.x

[B39] Broad GR Quicke DLJ The adaptive significance of host location by vibrational sounding in parasitoid wasps Proc R Soc Lond B Biol Sci 2000 267 1460 2403 2409 10.1098/rspb.2000.1298 PMC1690826 11133030

[B40] Vilhelmsen L Flexible ovipositor sheats in parasitoid Hymenoptera (Insecta) Arthropod Struct Dev 2003 32 277 287 10.1016/S1467-8039(03)00045-8 18089012

[B41] Godfray HCJ Parasitoids: behavioral and evolutionary ecology 1994 Princeton: Princeton University Press

[B42] Blomberg SP Garland TJ Ives AR Testing for phylogenetic signal in comparative data: behavioral traits are more labile Evolution 2003 57 4 717 745 12778543 10.1111/j.0014-3820.2003.tb00285.x

[B43] Gittleman JL Anderson CG Kot M Luh H-K Martins EP Phylogenetic lability and rates of evolution: a comparison of behavioral, morphological and life history traits Phylogenies and the comparative method in animal behavior 1996 Oxford, U.K: Oxford University Press 166 205

[B44] Segura DF Viscarret MM Paladino LZC Ovruski SM Cladera JL Role of visual information and learning in habitat selection by a generalist parasitoid foraging for concealed hosts Anim Behav 2007 74 131 142 10.1016/j.anbehav.2006.12.005

[B45] Gandolfi M Mattiacci L Dorn S Preimaginal learning determines adult response to chemical stimuli in a parasitic wasp Proc R Soc Lond B Biol Sci 2003 270 2623 2629 10.1098/rspb.2003.2541 PMC1691550 14728786

[B46] Rougerie R Smith MA Fernandez-Triana J Lopez-Vaamonde C Ratnasingham S Hebert PDN Molecular analysis of parasitoid linkages (MAPL): gut contents of adult parasitoid wasps reveal larval host Mol Ecol 2011 20 179 186 10.1111/j.1365-294X.2010.04918.x 21083857

[B47] Hinz R The biology of the European species of the genus *Ichneumon* and related species (Hymyenoptera: Ichneumonidae) Contrib Am Entomol Inst 1983 20 151 152

[B48] Folmer O Black M Hoeh W Lutz R Vrijenhoek R DNA primers for amplification of mitochondrial cytochrome c oxidase subunit I from diverse metazoan invertebrates Mol Mar Biol Biotechnol 1994 3 5 294 299 7881515

[B49] Smith PT Kambhampati S Völkl W Mackauer M A phylogeny of aphid parasitoids (Hymenoptera: Braconidae: Aphidiinae) inferred from mitochondrial NADH1 dehydrogenase gene sequence Mol Phylogenet Evol 1999 11 2 236 245 10.1006/mpev.1998.0575 10191068

[B50] Belshaw R Quicke DLJ A molecular phylogeny of the Aphidiinae (Hymenoptera: Braconidae) Mol Phylogenet Evol 1997 7 3 281 293 10.1006/mpev.1996.0400 9187088

[B51] Mardulyn P Whitfield JB Phylogenetic signal in the COI, 16S, and 28S genes for inferring relationships among genera of Microgastrinae (Hymenoptera; Braconidae): Evidence of a high diversification rate in this group of parasitoids Mol Phylogenet Evol 1999 12 3 282 294 10.1006/mpev.1999.0618 10413623

[B52] Thompson JN Higgins DG Gibson TJ CLUSTAL W: improving the sensitivity of progressive multiple sequence alignment through sequence weighting, position-specific gap penalties and weight matrix choice Nucleic Acids Res 1994 22 22 4673 4680 10.1093/nar/22.22.4673 7984417 PMC308517

[B53] Tamura K Dudley J Nei M Kumar S MEGA4: Molecular Evolutionary Genetics Analysis (MEGA) software version 4.0 Mol Biol Evol 2007 24 8 1596 1599 10.1093/molbev/msm092 17488738

[B54] Gillespie JJ Yoder MJ Wharton RA Predicted secondary structure for 28S and 18S rRNA from Ichneumonoidea (Insecta: Hymenoptera: Apocrita): impact on sequence alignment and phylogeny estimation J Mol Evol 2005 61 114 137 10.1007/s00239-004-0246-x 16059751

[B55] Swofford DL PAUP*. Phylogenetic analysis using parsimony (*and other methods). In: Version 4 2002 Sunderland, Massachusetts: Sinauer Associates

[B56] Nylander JAA MrModeltest v2 Program distributed by the author 2004 Uppsala: Evolutionary Biology Centre, Uppsala University

[B57] Posada D Buckley TR Model selection and model averaging in phylogenetics: advantages of Akaike information criterion and Bayesian approaches over likelihood ratio tests Syst Biol 2004 53 5 793 808 10.1080/10635150490522304 15545256

[B58] Brandley MC Schmitz A Reeder TW Partitioned Bayesian analyses, partition choice, and the phylogenetic relationships of scincid lizards Syst Biol 2005 54 3 373 390 10.1080/10635150590946808 16012105

[B59] Brown JM Lemmon AR The importance of data partitioning and the utility of Bayes factors in Bayesian phylogenetics Syst Biol 2007 56 4 643 655 10.1080/10635150701546249 17661232

[B60] Ronquist F Huelsenbeck JP MrBayes 3: Bayesian phylogenetic inference under mixed models Bioinformatics 2003 19 12 1572 1574 10.1093/bioinformatics/btg180 12912839

[B61] Ronquist F Larget B Huelsenbeck JP Kadane JB Simon D van der Mark P Comment on “Phylogenetic MCMC algorithms are misleading on mixtures of trees” Science 2006 312 367a 10.1126/science.1123622 16627724

[B62] Nylander JAA Ronquist F Huelsenbeck JP Nieves-Aldrey JL Bayesian phylogenetic analysis of combined data Syst Biol 2004 53 1 47 67 10.1080/10635150490264699 14965900

[B63] Stamatakis A Hoover P Rougemont J A rapid bootstrap algorithm for the RAxML web servers Syst Biol 2008 57 5 758 771 10.1080/10635150802429642 18853362

[B64] Yu DS Van Achterberg C Horstmann K DVD/CD T World Ichneumonoidea 2004 - Taxonomy, Biology, Morphology and Distribution 2005 Vancouver, Canada: http://www.taxapad.com

[B65] Carter DJ Hargreaves B A field guide to caterpillars of butterflies and moths in Britain and Europe 1986 London: William Collins Sons and Co. Ltd.

[B66] Lepidopterologen-Arbeitsgruppe Tagfalter und ihre Lebensräume, vol. Band 1 1994 Basel: Schweizerischer Bund für Naturschutz

[B67] Ebert G Die Schmetterlinge Baden-Württembergs. Band 1–10 1991–2005 Stuttgart: Eugen Ulmer GmbH und Co

[B68] Paradis E Claude J Strimmer K APE: analyses of phylogenetics and evolution in R language Bioinformatics 2004 20 289 290 10.1093/bioinformatics/btg412 14734327

[B69] R Development Core Team R: A language and environment for statistical computing 2009 Vienna, Austria: R Foundation for Statistical Computing

[B70] Pagel M Meade A Barker D Bayesian estimation of ancestral character states on phylogenies Syst Biol 2004 53 5 673 684 10.1080/10635150490522232 15545248

